# Determinants of self-rated health among highly educated Ukrainian women refugees in Czechia: analysis based on cross-sectional study in 2022

**DOI:** 10.1186/s12905-024-03053-8

**Published:** 2024-04-01

**Authors:** Ivana Kulhánová, Michala Lustigová, Dušan Drbohlav, Yana Leontiyeva, Dagmar Dzúrová

**Affiliations:** 1 Department of Social Geography and Regional Development, Faculty of Science, Charles University, Albertov 6, Prague, 128 00 Czechia; 2 Department of Demography and Geodemography, Faculty of Science, Charles University, Prague, Czechia; 3 Czech Social Science Data Archive, Institute of Sociology, Czech Academy of Sciences, Prague, Czechia

**Keywords:** Subjective health, War refugees, Social capital, Economic determinants, Systemic risks, Ukraine, Czechia

## Abstract

**Background:**

Russia’s military aggression against Ukraine set in motion a large number of refugees. Considerable amount of them came and stayed in Czechia. Refugees represent special vulnerable individuals often affected by war physically and psychologically. Due to the national regulations not allowing most of Ukrainian men aged 18–60 to leave the country, nowadays Ukrainian forced migration is relatively young and strongly gendered. Evidence suggests the higher probability for searching the safe refuge abroad among Ukrainian women with small children as well as those with relatively higher economic and cultural capital. The aim of this study is to identify the structural features of systemic risks associated with war migration by examining determinants of self-rated health among forcibly displaced highly educated Ukrainian women of productive age residing in Czechia.

**Methods:**

Data from one wave of the panel survey among Ukrainian refugees in Czechia conducted in September 2022 was used. Determinants of self-rated health including self-reported diseases and healthcare factors, lifestyle, human and social capital, economic factors, and migration characteristics were analysed using binary logistic regression.

**Results:**

About 45% highly educated Ukrainian women refugees in Czechia assessed their health as poor. The poor self-rated health was mostly associated with the number of diseases and depressive symptoms, and by social capital and economic factors. Having four and more diseases (OR = 13.26; 95%-CI: 5.61–31.35), showing some severe depressive symptoms (OR = 7.20; 95%-CI: 3.95–13.13), experiencing difficulties to seek help from others (OR = 2.25; 95%-CI: 1.20–4.23), living alone in a household (OR = 2.67; 95%-CI: 1.37–5.27), having severe material deprivation (OR = 2.70; 95%-CI: 1.35–5.41) and coming originally from the eastern part of Ukraine (OR = 2.96; 95%-CI: 1.34–6.55) increased the chance of these refugees to assess their health as poor.

**Conclusion:**

Social and economic determinants such as lack of social contacts for seeking help and material deprivation were found to be crucial for self-rated health and should be tackled via migration policies. Further, qualitative research is needed to better understand the mechanisms behind the factors affecting subjectively assessed health.

## Introduction

Russia’s military aggression against Ukraine, escalated on 24 February 2022, set in motion an exceptional and until then unprecedented wave of refugees in post-World War II Europe. These refugees came mainly to European Union countries, especially to geographically closer countries such as Poland, Slovakia, Czechia, but also to Germany or the UK [1]. Czechia became the country with one of the relatively highest number of refugees from Ukraine per capita partially also due to a long-term history of economic migration from Ukraine [2–4]. Available data suggest that significant part of Ukrainian refugees coming to Czechia were members of transnational families uniting with their family members already living (working) in Czechia [5]. Such a massive influx of war refugees, arriving in a very short time, has created enormous pressure on public services and state administration. European Union responded quickly and with relatively successful assistance. Thanks to the Temporary Protection Status Ukrainian refugees in Czechia and many other European countries gained in certain spheres even more rights than regular economic migrants. Despite intensive support provided to refugees upon arrival (like free access to the labour market, assistance with accommodation, early social support, full access to educational and healthcare system) the humanitarian and integration steps have not been and are not without problems and major challenges. Unfortunately, some of the bad practices and inequalities connected to the previous labour migration are reproduced when it comes to refugees.

It is estimated that approximately 350,000 Ukrainian refugees, holders of temporary protection status, were residing in Czechia by mid-July 2023 [6]. Czechia has a minimum of experience in assisting war refugees on its territory. The non-governmental sector, citizens and civil society and their solidarity play an important role in the overall successful management of the influx of Ukrainian refugees in Czechia.

War refugees represent a special group of vulnerable individuals who often lacked financial resources at their departure, were affected physically and psychologically by the effects of war, including death of family members, loss of housing, property or social network [7]. In addition, the perspective of war refugees largely differs from economic migrants in terms of so called “investment in integration” as it is often unclear whether they will remain in the destination country or return to Ukraine as soon as possible [8, 9]. Whereas there was research conducted on the integration of labour migrants from Ukraine and with Ukrainian unification of transnational families in Czechia [10, 11], more systematic and robust research related to Ukrainian war refugees in Czechia is still scarce, particularly when focused on individual level social, economic, and environmental factors affecting health. As it was already mentioned, family reunification played rather important role when it comes to the decision to seek refuge abroad [5]. Although, there is less evidence and literature on selectivity of refugee population, some studies suggest that even in forced migration self-selection (in terms of education, socioeconomic status and health) might play an important role [12]. There is evidence suggesting that the socioeconomic profile of Ukrainian refugees is different from internally displaced persons and that refugees do not necessarily represent poorest and the most vulnerable groups. On the contrary, they are often people with a relatively higher social and economic status, cultural capital, and ties abroad [13]. Most studies analysing health of Ukrainian refugees focused mainly on mental health [14–16]. There are only a few studies concentrating on self-rated health [17].

Self-rated health is determined by a wide range of factors such as demographic, social, economic, lifestyle [18–20]. In the case of refugees, factors influencing the migration process – such as reasons for migration, length of stay, refugees’ legal status, social network, previous experience of migration and also thoughts of return – may play an important role as well [21–23]. In terms of determinants of health, studies are mostly oriented towards migrants, not refugees [24–27]. Nevertheless, from the summaries of studies on migrants, some parallels can be drawn for the refugee group as well. Regarding the health status of migrants in the host country, the literature is largely focused on so called healthy migrant effect [28–32] referring to the health advantage of migrants when immigrants are on average healthier than the native born. Although the healthy migrant effect has not been found yet for refugees, it is assumed that the refugee population is also selective [12, 33], i.e., that only those people who can manage the journey can leave their homes, and this from many aspects, especially if they are physically mobile and have the inner strength to leave their homes.

The aim of this study was therefore to examine the self-rated health status of the Ukrainian refugees in Czechia and to analyse its determinants related to the self-reported diseases and healthcare factors, lifestyle, human and social capital, and economic characteristics using unique data on Ukrainian refugees in Czechia collected in September 2022. In addition, the role of geographical context of the place of origin in Ukraine on self-rated health was investigated.

## Methods

### Data collection

In response to the full-scale Russian invasion on Ukraine in February 2022 resulting in the large influx of Ukrainians to Czechia, the Institute of Sociology of the Czech Academy of Sciences in co-operation with PAQ Research agency launched a unique online panel survey of Ukrainian refugees focusing on different aspects of integration and everyday life. The panel was established in cooperation with the Ministry of Labour and Social Affairs of the Czech Republic, and it consists of Ukrainian refugees aged 18 and over who applied for humanitarian financial aid in March, April, and May 2022. The Ministry approached the benefit claimants with a brief census and an offer of entry to a panel survey administered by the Institute of Sociology. The subsequent panel survey was launched as separate research project not related to the ministerial census and all the respondents agreed with informed consent mentioning data confidentiality and anonymity of responses. All respondents identified were verified by phone during the recruitment phase. A sample of 3,082 respondents, each representing a separate household, was drawn. At the beginning of each data collection within this panel survey, respondents were asked to report whether they were still residing in Czechia. Between June 2022 and June 2023 six waves of this panel survey were conducted. This study used primarily the data from the third wave dedicated to physical and mental health of refugees collected between 6 and 22 of September 2022; although some of the indicators (like material deprivation index, language competences, housing conditions, social contacts, plans for return, education etc.) were used from the data of the previous waves. In total, 1,347 respondents participated in the mentioned wave. A slightly smaller response rate (comparing to two first waves of the study conducted in June/July 2022 and August 2022 – 1,700 and 2,351 respectively) could be accounted to the change in the refugee population but also to the sensitive nature of the topic. Participants were not compensated directly but a donation to a selected charity project assisting people affected by the war in Ukraine was offered on behalf of respondents for each completed questionnaire.

### Study population and measures

For the purpose of this study, we selected women with tertiary education as they accounted for most respondents (about 70%) and also restricted the sample to women at productive age (18–64 years). The resulting data was therefore based on the answers of 919 Ukrainian women refugees.

The perceived state of health of the Ukrainian refugees was based on the question “How do you rate your overall state of health?” with five possible answers – very good, good, neither good nor bad, bad, very bad. For the analyses, we dichotomized the perceived state of health as good (very good, good) and poor (neither good nor bad, bad, very bad). The outcome was defined as reporting poor health.

As we selected highly educated women, the sociodemographic variables used in the analysis were merely age applied as a continuous variable from age 18 to 64 years.

Based on the framework of Dahlgren and Whitehead [34] and Marmot and Wilkinson [35] determinants were grouped into five domains: (1) self-reported diseases and healthcare factors (number of barriers to healthcare access, number of diseases, lack of medicine, depression symptoms); (2) lifestyle (smoking, alcohol, body mass index); (3) social factors (close people, interest of others, get help, children in household, contact in Czechia prior to arrival, household size, knowledge of language, housing quality); (4) economic factors (job, material deprivation); (5) migration characteristics (return intentions, arrival time, husband in Ukraine, and geographical region).

Self-reported diseases and healthcare factors variables included the number of diseases, the number of barriers to healthcare access, lack of medicines and depressive symptoms. The number of diseases was determined with a set of questions asking whether respondent suffered from 12 listed (plus any other opened to specify) diseases during the past 12 months, including high blood pressure, heart attack, stroke, high level of cholesterol, diabetes, cancer, chronic respiratory diseases, depression and anxiety, diseases of the spine, covid (or post-covid), HIV/AIDS, tuberculosis, and other. The number of diseases was created as a sum of given comorbidities and categories into five groups: (1) no disease, (2) 1 disease, (3) 2 diseases, (4) 3 diseases, and (5) 4 and more diseases. The number of barriers to healthcare access was assessed by asking question “Did you encounter the following barriers when looking for a doctor in Czechia?”. The four following barriers were considered – (1) language barrier, (2) respondent does not know how the system works in Czechia, (3) respondent does not know what to do, how and where to register, and (4) respondent does not know if he/she would have to pay for it. The number of barriers were summed up and the combination of given barriers divided into four groups: (1) no barriers, (2) 1 barrier, (3) 2 barriers, and (4) 3 and 4 barriers. The number of lacking medicines was defined from question “Do you access to all medicines, and do you use them also in Czechia?” including medicines for diseases indicated above. This variable was dichotomized including (1) no lack of medicines, and (2) 1 and more lacking medicines that needed. Depressive symptoms were assessed by a widely used Patient health questionnaire depression scale (PHQ-8) related to selected depressive symptoms in the last 14 days, which assesses depressive symptoms in individuals. The PHQ-8 scale is the sum of the 8 items and measure of current depressive symptoms and the maximum score of PHQ-8 is 24 points. More information on PHQ-8 scale tool can be found in literature [36]. Ukrainian version of PHQ-8 scale used in the survey was proven to have a good internal consistency (Cronbach’s alpha = 0.87). The study conducted in 2023 suggests a high prevalence of and a strong link between symptoms of depression and anxiety among Ukrainian refugees in Czechia accompanied by a very low help-seeking [37]. Based on the given score, the depressive symptoms variable was categorized into four groups: (1) no depression (0–4 points), (2) mild depression (5–9 points), (3) medium depression (10–14 points), and (4) severe depression (15 + points).

Lifestyle variables included smoking, alcohol consumption, and body mass index (BMI). Smoking status was assessed by asking question “Do you currently smoke any tobacco products? If yes, how often?” Women were divided into three categories according to the smoking status: (1) smoke daily, (2) smoke occasionally, and (3) never. Alcohol consumption was measured combining two questions “How often do you drink alcoholic beverages?” and “When you drink an alcoholic beverage, how many glasses do you usually drink?”. Women were divided according to the alcohol consumption into three groups: (1) do not drink alcohol, (2) 1–2 glasses of alcohol, (3) 3 and more glasses of alcohol. BMI was measured based on self-reported height in cm and weight in kg and categorized into four groups defined by the WHO: 1) underweight (BMI < 18.5 kg/m^2^), healthy weight (BMI > = 18.5 kg/m^2^ & BMI < 25 kg/m^2^), overweight (BMI > = 25 kg/m^2^ & BMI < 30 kg/m^2^), obesity (BMI > = 30 kg/m^2^).

Human and social capital variables were represented by the number of close persons, interest of others, ability to get help if needed, household size, contact persons in Czechia prior arrival, knowledge of Czech language and housing quality. The number of close persons was assessed by asking question “How many people are so close to you that you can count on them in case of serious personal problems?”. According to the indicated number of persons, women were divided into three categories: (1) no close person, (2) 1–2 close persons, (3) 3 and more close persons. Interest of other people was measured by question “How much are other people around you interested in what you do? What interest do they show?”. Women were grouped into three groups based on perception of interest shown from other people: (1) great interest and some interest, (2) neither great nor little interest, and (3) only little and no interest at all. The ability to get help if needed was assessed by asking question “How easy it is for you to get help from other people if you need it?” Based on provided answers women were divided into three groups: (1) very easy and easy to get help, (2) possible, and (3) difficult and very difficult to get help. The indicator for the household type was constructed from a set of questions in the household grid (number of household members, age of given household member and his or her relationship to the respondent). Based on the answers the respondents were divided into four groups: (1) single member, (2) two and more members without children, (3) single parent with children, and (4) family with children. Social contacts in Czechia prior to arrival were measured by asking question “Did you have any contacts with people living in Czechia before your arrival?” including family members and relatives, other Ukrainian and Czechs. Here women were divided into two groups: (1) with previous contacts, and (2) no previous contacts. Knowledge of Czech language was determined based on question “What are your language skills in Czech language?” based on language proficiency according to the Common European Framework of Reference for Languages (CEFR) organized in six levels from A1 to C2. Women were divided into three groups according to their language knowledge: (1) level A1 and lower, (2) level A2, (3) level B1 and higher. Housing quality was assessed by asking question “Where do you currently live?” grouping women into three categories: (1) lodging house, (2) rental accommodation, (3) other, including solidarity housing.

Economic characteristics were assessed by economic status and material deprivation. Women activity at the labour market was asked by question “Are you currently in paid work?” including job in Czechia or remotely in Ukraine or elsewhere abroad. Women were divided into two groups according to their labour status: (1) yes, or (2) no. For the measurement of material deprivation, we used the adopted EU indicator constructed from 13-item set of questions [38]. The scale was measuring if the household could afford covering selected everyday and extraordinary expenses. The respondents were asked if their households could afford covering 13 types of expenses (listed in rotation): (1) some unexpected expenses of approx. 500 Euros, (2) paying annually for at least a week’s vacation outside your current Czechia home for all household members, (3) eating meat, poultry, or fish (or their vegetarian replacements) at least every other day, (4) heating the flat sufficiently, (5) replacing worn furniture with new one, (6) replacing worn-out clothes with at least some new pieces (not second-hand), (7) having at least two pairs of well-fitting shoes for each household member, (8) meeting with friends at a café, restaurant, bar, or at home several times a month, (9) regularly engage in a paid leisure activity (sports, going to the cinema, etc.), (10) spending a set amount on yourself every week (e.g. a cinema ticket, a small gift, etc.), 11) using a private car, 12) paying for your housing costs (rent, utilities, etc.), and 13) paying for internet access. Based on provided answers women were divided into three groups according to their level of deprivation: (1) no material deprivation, (2) some material deprivation, and (3) severe material deprivation. Those who could not cover up to 4 out of the 13 mentioned expenses were described as those not suffering from material deprivation; respondents who could not afford 7 and more expenses were treated as those with severe material deprivation.

Migration-related variables included plans for return to Ukraine within next 2 years, time of arrival in Czechia, if women had husband/partner in Ukraine, and geographical region of origin. Return to Ukraine was asked by question “Do you want to return to Ukraine within the next 2 years?” and divided in three groups: (1) with plans for return (definitively and rather want to return), (2) with no plans for return (definitively and rather want to stay abroad, in Czechia or other country), and (3) not decided (do not know or not sure). Time of arrival to Czechia was assessed by asking question “When did you come to Czechia?” and grouped into two categories: (1) February-March 2022, and (2) April-June 2022. The series of questions concerning close family members who stayed in Ukraine were used to calculate the predicator signifying women had spouse or partner left back home. Here we differentiated only those who mentioned that their spouse or partner stayed back in Ukraine from the rest, i.e., we combined women with no partner with those who had partner in other country abroad. Geographic region of origin was determined based on question “In which region of Ukraine did you live for a long time before coming to Czechia?”. The 25 regions of Ukraine were divided into four main geographical areas – West (regions of Volyn, Zakarpattia, Ivano-Frankivsk, Lviv, Rivne, Ternopil, Khmelnytskyi, Chernivtsi), Center (regions of Kyiv City, Vinnytsia, Zhytomyr, Kyiv, Kirovograd, Poltava, Sumy, Cherkasy, Chernihiv), South (regions of Dnipropetrovsk, Zaporizhia, Mykolaiv, Odesa, Kherson) and East (regions of Donetsk, Luhansk, Kharkiv).

### Statistical analysis

Characteristics of the Ukrainian women refugees were illustrated using descriptive statistics as absolute numbers and proportions. The relationship between self-rated health and self-reported diseases and healthcare factors, lifestyle, social, economic, and migration factors was analysed using binary logistic regression. The results were presented as odds ratios (OR) with the corresponding 95% confidence intervals (95%-CI) and p-value. All the tests were conducted at a significance level of 0.05. The analyses were performed in the statistical software STATA 17.

## Results

A total of 919 highly educated women age of 18–64 years were included in the study. About 55% of these women (*N* = 507) reported their health as “good” or “very good”. The characteristics of respondents are described in Table 1.

**Table 1:** Characteristics of respondents, Ukrainian women refugees, 18–64 years, Czechia

Determinant	Number of respondents	Share (in %)
**Self-rated health (N = 919)**		
good	507	(55.2%)
poor	412	(44.8%)
**Age (N = 919), Mean (SD)**	37.8	(8.7)
**Healthcare factors**		
**Number of barriers to healthcare access (N = 919)**		
0	347	(37.7%)
1	306	(33.3%)
2	134	(14.6%)
3+	132	(14.4%)
**Number of diseases (N = 919)**		
0	183	(19.9%)
1	295	(32.1%)
2	237	(25.8%)
3	122	(13.3%)
4+	82	(8.9%)
**Number of lacking medicine (N = 919)**		
0	810	(88.1%)
1+	109	(11.9%)
**Depressive symptoms (N = 860)**		
no depressive symptoms	222	(25.8%)
mild depressive symptoms	275	(32.0%)
moderate depressive symptoms	182	(21.2%)
severe depressive symptoms	181	(21.0%)
**Lifestyle factors**		
**Smoking status (N = 918)**		
daily	117	(12.8%)
occasionally	127	(13.8%)
never	674	(73.4%)
**Alcohol consumption per week (N = 914)**		
never	174	(19.0%)
1–2 glasses	680	(74.4%)
3 + glasses	60	(6.6%)
**BMI category (N = 896)**		
underweight	63	(7.0%)
healthy weight	546	(60.9%)
overweight	203	(22.7%)
obesity	84	(9.3%)
**Human and social capital**		
**Number of close persons (N = 913)**		
0	156	(17.1%)
1–2	591	(64.7%)
3+	166	(18.2%)
**Interest of others (N = 894)**		
yes	553	(61.8%)
neither-nor	149	(16.7%)
no	192	(21.5%)
**Get help (N = 885)**		
easy	189	(21.4%)
possible	479	(54.1%)
difficult	217	(24.5%)
**Household size (N = 891)**		
1 member	102	(11.4%)
2 + members without children	115	(12.9%)
Single parent with children	367	(41.2%)
Family with children	307	(34.5%)
**Contact in Czechia prior arrival (N = 919)**		
yes	596	(35.2%)
no	323	(64.8%)
**Knowledge of Czech language (N = 919)**		
A1-	367	(39.9%)
A2	396	(43.1%)
B1+	156	(17.0%)
**Housing quality (N = 918)**		
lodging house	195	(21.2%)
rental accommodation	231	(25.2%)
other	492	(53.6%)
**Economic characteristics**		
**Job in Ukraine or in Czechia (N = 919)**		
yes	388	(42.2%)
no	531	(57.8%)
**Material deprivation (N = 891)**		
no	100	(11.2%)
yes, moderate	165	(18.5%)
yes, severe	626	(70.3%)
**Migration characteristics**		
**Return to Ukraine within 2 years (N = 891)**		
yes	462	(51.9%)
no	396	(44.4%)
do not know	33	(3.7%)
**Arrival to Czechia (N = 919)**		
February-March 2022	775	(84.3%)
April-June 2022	144	(15.7%)
**Husband in Ukraine (N = 891)**		
yes	548	(61.5%)
no / not applicable	343	(38.5%)
**Geographical region of origin (N = 915)**		
West	82	(9.0%)
Central	348	(38.0%)
South	276	(30.2%)
East	209	(22.8%)

*Note* Prevalence (%) for categorical and mean (SD) for continuous variables

The average age of Ukrainian women refugees was about 38 years. Regarding the self-reported diseases and healthcare factors, about 38% of Ukrainian women in the dataset reported no barriers to healthcare access and about 20% of them left Ukraine without any disease. Almost 88% of women in the data indicated that they do not lack any medicine. Regarding depression about 26% of women did not report any depressive symptoms, in contrast to 21% of women who reported severe depressive symptoms. In terms of lifestyle, highly educated Ukrainian women refugees showed relatively healthy lifestyle, about 73% were non-smokers, 19% did not drink alcohol and only 9% were obese. Regarding social factors, 62% indicated that they feel interest of others, 75% can get help easily or possibly, and 35% had a contact in Czechia prior arrival. About 76% were women with children in the household. However, only 17% of women had advanced knowledge of Czech language (B1+). Out of economic factors, more than half of women (58%) had no job in Czechia or in Ukraine and 70% seem to suffer from severe material deprivation. With regard to migration characteristics, more than half of women shortly upon their arrival expressed the wish to return to Ukraine within next 2 years. Thought it is worth mentioning that this indicator is from the first wave of the panel survey conducted in June 2022, which is 3 months before the wave on health aspects. The majority of women (85%) arrived in Czechia between February and March 2022, 62% had a husband in Ukraine and more than half of the Ukrainian women came from South or East of Ukraine, the regions suffering the most from Russian military aggression.

The results of binary logistic regression are presented in Table 2. Age was confirmed as a significant variable influencing the self-rated health. The chance of poor self-rated health increases with age. As it was expected, the declaration of poor health was by far the most strongly associated with the number of declared diseases by the respondent and the severity of depressive symptoms as measured by the PHQ-8 severity scores. The odds ratio (OR) for poor self-rated health increased with the number of diseases and severity of depressive symptoms. Ukrainian women refugees who declared four or more diseases had more than 13 times higher chance to assess their health as poor compared to their counterparts with no disease (OR = 13.26; 95%-CI: 5.61–31.13). Regarding the depressive symptoms, a woman with the most severe degree of depressive symptoms was more than 7 times worse off in terms of self-rated health compared to a woman without depressive symptoms (OR = 7.20; 95%-CI: 3.95–13.13).

**Table 2:** Impact of determinants on poor self-rated health, Ukrainian women refugees, 18–64 years, Czechia

Determinant	Adj. OR	95%-CI	p-value
**Age**	1.04	(1.02–1.06)	0.001
**Healthcare factors**			
**Number of barriers to healthcare access**			
0	1		
1	1.05	(0.67–1.63)	0.843
2	1.50	(0.85–2.63)	0.162
3+	1.64	(0.92–2.92)	0.095
**Number of diseases**			
0	1		
1	1.45	(0.81–2.58)	0.209
2	3.68	(2.03–6.66)	< 0.001
3	7.62	(3.77–15.41)	< 0.001
4+	13.26	(5.61–31.35)	< 0.001
**Number of lacking medicines**			
0	1		
1+	1.21	(0.66–2.22)	0.531
**Depressive symptoms**			
no depressive symptoms	1		
mild depressive symptoms	1.79	(1.06–3.04)	0.030
moderate depressive symptoms	2.96	(1.65–5.31)	< 0.001
severe depressive symptoms	7.20	(3.95–13.13)	< 0.001
**Lifestyle factors**			
**Smoking status**			
daily	1.10	(0.61–1.97)	0.756
occasionally	0.75	(0.44–1.28)	0.293
never	1		
**Alcohol consumption per week**			
never	1		
1–2 glasses	0.65	(0.40–1.05)	0.076
3 + glasses	0.94	(0.40–2.19)	0.877
**BMI category**			
underweight	2.41	(1.09–5.33)	0.030
healthy weight	1		
overweight	0.77	(0.49–1.20)	0.251
obesity	1.71	(0.90–3.25)	0.104
**Human and social capital**			
**Number of close persons**			
0	1		
1–2	2.78	(1.56–4.97)	0.001
3+	2.29	(1.10–4.79)	0.027
**Interest of others**			
yes	1		
neither-nor	0.68	(0.40–1.16)	0.160
no	1.57	(0.92–2.67)	0.098
**Get help**			
easy	1		
possible	1.37	(0.83–2.24)	0.217
difficult	2.25	(1.20–4.23)	0.012
**Contact in Czechia prior arrival**			
yes	1		
no	1.04	(0.70–1.55)	0.837
**Household size**			
1 member	2.67	(1.37–5.27)	0.004
2 + members without children	1.21	(0.66–2.22)	0.547
Single parent with children	1.36	(0.88–2.08)	0.164
Family with children	1		
**Knowledge of Czech language**			
A1-	1.26	(0.71–2.24)	0.437
A2	1.03	(0.58–1.81)	0.921
B1+	1		
**Housing quality**			
lodging house	0.90	(0.53–1.55)	0.714
rental accommodation	1		
other	0.95	(0.61–1.48)	0.805
**Economic factors**			
**Job in Ukraine or in Czechia**			
yes	1		
no	0.66	(0.45–0.96)	0.031
**Material deprivation**			
no	1		
yes, moderate	2.53	(1.19–5.39)	0.016
yes, severe	2.70	(1.35–5.41)	0.005
**Migration characteristics**			
**Return to Ukraine within 2 years**			
yes	1		
no	0.81	(0.55–1.19)	0.278
do not know	1.27	(0.47–3.42)	0.633
**Arrival to Czechia**			
February-March 2022	1		
April-June 2022	1.50	(0.90–2.50)	0.121
**Husband in Ukraine**			
yes	1		
no / not applicable	1.40	(0.95–2.07)	0.094
**Region**			
West	1		
Central	1.72	(0.82–3.65)	0.154
South	1.77	(0.83–3.79)	0.142
East	2.96	(1.34–6.55)	0.007

*Note* Odds ratio adjusted for all determinants

Unlike the number of diseases and depressive symptoms, lifestyle did not seem to play an important role for the self-rated health among Ukrainian women refugees. The only significant differences were found for underweighted Ukrainian women refugees who had 2.4 times higher chance (OR = 2.41; 95%CI: 1.09–5.33) to declare poor self-rated health compared to women with healthy weight.

Concerning the human and social capital, here the findings are not straightforward. The data did not confirm the expected positive effect of having close persons on whom an individual can count in case of serious personal problems on subjective health assessment. On the contrary, women having one or two close persons (OR = 2.78; 95%-CI: 1.56–4.97) and women having three and more close persons (OR = 2.29; 95%-CI: 1.10–4.79) had about 2.8 times and 2.3 times, respectively, higher chance to assess their health as poor compared to women with no close persons. Thought, important predictor of self-rated health was found to be opportunities to get help from others. Women for who was difficult to get help from others had 2.3 times higher chance to assess their health as poor compared to women who were able to get help from others easily (OR = 2.25; 95%-CI: 1.20–4.23).

Regarding economic factors, the relationship between health and material deprivation was proved significant. Women who faced a severe material deprivation had 2.7 times higher chance of poor self-rated health compared to those who were not materially deprived (OR = 2.70; 95%-CI: 1.35–5.41). Based on the results, women with no paid job in Czechia or remotely in Ukraine were found to have lower chance of poor self-rated health (OR = 0.66; 95%-CI: 0.45–0.96).

Migration characteristics were not found significantly associated with poor self-rated health, except the region of origin. Women coming to Czechia from the Eastern Ukraine region (regions of Donetsk, Luhansk, Kharkiv) had almost 3 times higher chance to declare poor health than women who came to Czechia from the West region (regions of Volyn, Zakarpattia, Ivano-Frankivsk, Lviv, Rivne, Ternopil, Khmelnytskyi, Chernivtsi) (OR = 2.96; 95%-CI: 1.34–6.55).

## Discussion

### Main findings

The number of diseases and depressive symptoms, social capital (difficulties with getting necessary help and single person household) and economic factors (having job and material deprivation) were found to be crucial predictors of self-rated health of Ukrainian women refugees. Furthermore, women coming from eastern part of Ukraine to Czechia reported remarkably higher chance of poor subjectively assessed health compared to their counterparts coming from the western parts of Ukraine. In contrast, in our data we did not find enough evidence to support the assumption of the effect of lifestyle on self-rated health among highly educated Ukrainian women refugees.

### Interpretation, discussion

In this study, we characterized some of the salient features of self-rated health in relation to selected determinants such as self-reported diseases and healthcare factors, lifestyle, human and social capital, economic situation, and migration characteristics among highly educated Ukrainian women refugees in Czechia.

Studies that addressed international migration in focus to self-rated health in Czechia are quite few [11, 17, 20, 39] and most of them are limited to economic labour migration. However, international literature reported different consequences of economic migration, which is predominantly voluntary, from the refugee migration, which is to a great extent forced [40–43]. Whereas voluntary migration is mainly driven by economic cost-benefit factors of the migrants, forced migration is the result of circumstances that are mostly outside the control of the migrants [43]. The refugees often experienced traumatic situations not only due to war but also as a consequence of travelling and temporary living conditions in refugee camps [40]. Economic migrants decide to relocate based on the relative opportunities abroad while refugees are forced to unexpectedly migrate due to vulnerability to persecution [40]. Therefore, it is necessary to keep in mind that refugees are not economically selected to the same degree as economic migrants. As a consequence, refugees often arrive in a host country not ready at all for migration and for using their human capital, including knowledge of the local environment and language, compared to traditional economic labour migrants [40]. From this point of view, it is important to note that our results may slightly differ from findings of other studies that focused more on economic migration.

Health status and lifestyle itself have important implications for self-rated health. An association between self-rated health and chronic diseases, disability and depressive symptoms were found in numerous studies [44–46]. This is in line with our results as we have found that Ukrainian women refugees had higher chance to declare poor health with increasing number of diseases and severity of depressive symptoms.

Although international studies reported that poor self-rated health is often associated with less physical activity, smoking behaviour and obesity [47–50], we did not find any significant effect of lifestyle indicators measured by current smoking status, alcohol consumption and body mass index category (BMI) and self-rated health. This could be partially the effect of our target group (highly educated women in productive age), which in general has rather healthy lifestyle. Though, underweight appeared to be certain exception. Underweighted women refugees had higher chance to declare poor health compared to those with healthy weight.

Most of previous studies focused merely on lifestyle neglecting economic or social characteristics [47–50]. Based on our results, it seems that other aspects than lifestyle such as e.g., human and social capital, or economic characteristics also play an important role in assessment of subjective health among refugees. We found that Ukrainian women refugees living alone had higher chance to assess poor subjective health compared to women living in whole families with children. Living alone is a predictor of poor self-rated health due to greater isolation, lack of close peer psychological support, fewer contacts to the outside environment including less information. The association of loneliness and poor self-rated health is well established in the previous studies [51–53]. Feeling lonely may well lead to development of depressive symptoms, especially among older migrants [53].

Perception of health is also based on human and social capital. In terms of human capital, language proficiency of the destination country of migration was expected to be associated with self-rated health [54, 55] as migrants with language knowledge may be better able to navigate in the society, make better use of healthcare system and find easier a job. However, our study did not find statistically significant relationship between self-rated health and the knowledge of Czech language. The explanation can probably be found in the fact that the huge Ukrainian diaspora that arrived in a large number even before the Russian aggression was and is to a large extent able to effectively help those who do not speak the language to orient themselves and to function in Czech society without major handicaps [2]. Apart from that, the proximity of Slavic languages may also help Ukrainian refugees to reasonably communicate and to learn Czech quickly [56]. On the other side, the language proficiency of most of the refugees just couple of months after arrival is in general rather low. Therefore, even those respondents in our survey, who were identified as those with relatively better language skills (our reference group with B1 + level constitutes only 17% of respondents) might not be at a great advantage and they might also lack specific vocabulary when it comes to the conversation with doctors.

In contrast to human capital, social capital was found to be an essential predictor of self-rated health among highly educated Ukrainian women refugees. The refugees, who stated that it is easy for them to get needed help from other people, had significantly better health assessment. Social networks have been discussed as a main determinant of self-rated health in several international publications [57–59]. Good relationships and social support improve health in general, both directly and indirectly, with social support buffering the effects of various stressors [60, 61]. Feelings of belonging, recognition, care, and communication have protective effect on health [35]. Social exclusion has a direct effect on health, it also affects health through a less accessible health system, and it also has an indirect effect on health through its impact on other determinants [62]. Conversely, higher levels of social integration are positively associated with migrants’ health [63–65].

Paradoxically, a different result is yielded by linking self-rated health to the number of close people. The likelihood of declaring poor self-rated health increases with the number of people close enough that the individual can count on them in case of serious problems. Although the questions do not identify more precisely whether these are support networks made up of Czechs or Ukrainian compatriots, it is suggested that in this case weak ties (created with acquaintances with whom we have less powerful connections) rather than strong ties (people we know well, trust, and speak to them often) are more likely to be promoted [66–68]. Other possible explanation can be related to the fact that different social classes have access to different sets of resources. Resources from family and friends from intermediate and higher services classes are beneficial for self-rated health whereas resources of family and friends from working class appear to be rather detrimental for self-rated health [69]. Thus, somewhat surprisingly, Czechs are probably more supportive of refugees than other Ukrainians. This only demonstrates again the importance of the Czech public overwhelming active solidarity towards the victims of Russian aggression, which is omnipresent. This is also in line with results from an analysis of a different research sample of Ukrainian refugees and a differently designed research in relation to the labour market: “Contact with family members or compatriots turned out to be less significant than contacts with friends and acquaintances of Czech origin in terms of their helpfulness in involving refugees in the labour market” [9].

Self-rated health differs among refugees according to their economic characteristics. Labour market failure, unemployment, and job insecurity are often seen as health risks as they lead to increased stress, i.e., disrupted mental health, poorer subjectively perceived health, increased morbidity or risk of premature death [35, 70]. There are two essential aspects of the economic dimension: (1) material deprivation and (2) an active role at the labour market. In available literature material derivation was found to be an important predictor of self-rated health [71, 72]. In our data, Ukrainian women refugees who suffered from severe material deprivation showed the highest chance to declare poor health. Their very limited financial and material resources prevent them from making full or effective use of some health-rehabilitation and social services, obtaining medicines with a financial supplement, or even moving to a more convenient region or location in Czechia.

Obtaining an adequate job should contribute to a better subjective perception of one’s own health due to increased self-esteem, increased income, greater contact with the majority society [20]. In our study, however, working refugees currently performing paid work either in Czechia or remotely in Ukraine or elsewhere abroad had higher chance to assess their health as poor compared to the women who did not have paid work. The reason may be due to our specific research sample consisting of women with university degree. Based on their educational qualification, they may encounter mismatch of their qualification with offered jobs, especially due to lack of Czech language proficiency or transferability of university degrees, which are often manually, not intellectually demanding and do not correspond with their education or expectations leading to dissatisfaction or even frustration due to human capital loss [3, 73]. Over-qualification has already been reported to be harmful for self-rated health [74, 75] and mental health [76]. Some migrants may also suffer from discrimination at workplace deteriorating their self-rated health [77].

Refugees often face an uncertain future. According to our results, more than half of the Ukrainian women refugees indicated that they would like to return to Ukraine when the situation in their country allows. These women do not have the pressure to integrate in the Czech society with learning the language or searching for a job as they have different priorities and as they expect to soon return back to Ukraine [42]. In addition, these women are often accompanied by a child or more children and their priority is to take care of them instead of search for employment. Limited social networks, combined with child/children care (pre-school or compulsory school) often prevent mother from being fully active in the labour market. Still more than half of these women wish to return to Ukraine soon and therefore the integration at labour market is also slower. Although Ukrainian refugee have the advantage of free access to the labour market (unlike many other labour migrants), there are many additional pitfalls to their successful incorporation into the labour market. Factors preventing successful integration can vary from the pour language skills and lack of experience or social capital to the mental health problems, traumatic experiences, life uncertainties, family hardships, discrimination on the labour market and low motivations to invest in skill recognition and search of the “proper job”.

Perception of health among refugees may depend on the previous experience with war which could be partially measured by the geographical place of origin. We found that Ukrainian women refugees coming from the eastern part of Ukraine (regions of Donetsk, Luhansk, Kharkiv) where the population experience the heaviest military aggression had 3 times higher chance to assess their health as poor compared to their counterparts coming from the western part of Ukraine. Experiencing a war and the intensity of fighting had mental health consequences [78]. Women from the eastern part of Ukraine where the threats for lives due to armed conflict are the greatest were affected physically and psychologically carrying their war trauma to the host country whereas women coming from the western part who were though affected by the fear of imminent armed conflict, however, did not experience the direct effects of the war. The fact that refugee women from the western regions of Ukraine had to take a more difficult migratory route to Czechia than women from the western regions of Ukraine, geographically closer to Czechia, certainly plays an important role. This different perception of war in the geographical context of Ukraine and the reasons behind when fleeing from Ukraine could play an important role in assessment of subjective health after arriving in Czechia. In addition, refugees who are unable or unwilling to return home for fear may differ from refugees who can return home due to safer situation of their place as many refugees express their desire to return back to Ukraine [79]. Apart from the close geographical location, the historical context of the past interdependence of parts of western Ukraine with Czechoslovakia is also reflected in the traditionally more intense migration ties of these regions with Czechia. As empirically documented, Ukrainian refugees from Western Ukraine have higher employment rates in Czechia compared to refugees from other regions of Ukraine, and are also younger on average with lower educational attainment, better Czech language skills, and higher levels of contact in Czechia prior to arrival [9].

### Limitations

This study has its limitations. The sample is not fully representative in a statistical sense. We selected highly educated women aged 18–64 years for this study as the majority of survey respondents were women with tertiary education. We therefore analysed a particularly selective population. Despite the longitudinal character of the survey, this article is based on a cross-sectional data set from the third wave (with some indicators collected in the previous waves). The nature of available data does not allow us to track and evaluate trends over time or to derive any causal inferences about the direction of the relationship between the healthcare factors, lifestyle factors, social capital, economic, and migration trajectories and the self-rated health. We can only investigate if there is any association between the mentioned factors and the subjective health status. The refugees were captured very soon after their arrival in Czechia and thus the impact of new factors in the destination country on the self-rated health of refugee population might not be that obvious yet; a longer time period for the study is needed. Further, the respondents were recruited from the benefit claimants. Although at the time of the recruitment into the panel survey (June 2022) the most of the Ukrainian refugees (79%) with temporary protection regardless of their economic status did use the opportunity to receive financial aid from the Czech state to cover the basic needs [5], the selection process based on the ministerial data did not include those refugees who did not claim mentioned benefits. In addition, both the registration for the benefits and the data collection for the survey were conducted online, which allows to assume certain bias towards respondents with sufficient digital skills. At the same time, online mode and the anonymity of the survey suggests the lower susceptibility to social desirability bias in provided responses. The participation in the survey was compensated in a form of a donation to the list of charities assisting people affected by the war in Ukraine. From this point of view, the social desirability bias could also occur. This bias refers to the likelihood of underreporting what could be considered as undesirable behaviour or on contrary overreporting the desirable behaviour. Although the respondents were asked sensitive questions about their e.g. mental health, we believe that the social desirability bias was reduced by assuring the anonymity and the confidentiality of responses and by explaining the importance of correct answers for designing suitable policies in the field of medical and psychological care to Ukrainian refugees.

## Conclusion

Apart from the number of diseases and depressive symptoms, social capital and economic factors found to be the essential determinants of self-rated health among Ukrainian women refugees. Not only women with higher number of diseases, but also those indicating severe depressive symptoms, living alone in a household, reporting difficulties seeking help from others, and suffering from material deprivation appears to be the most vulnerable group when it comes to subjective health assessment. To improve self-rated health, it is necessary to focus on accessibility of healthcare, to support socialization of refugees and to improve their economic situation and labour market integration. For the further research on subjectively assessed health, it would also be beneficial to apply a qualitative approach to better capture and understand the reasons behind the self-reported health problems among Ukrainian women refugees and to clarify many nuances and refugee contexts.

## Data Availability

This publication used data acquired through the data services of the Czech Social Science Data Archive/European Social Survey – Czech Republic (CSDA/ESS-CZ). The CSDA/ESS-CZ research infrastructure project is supported by the Ministry of Education, Youth and Sports within the framework of grant LM2023046. Data will be publicly available at the data archive in the half of 2024.

## Abbreviations

BMI: Body mass index
CEFR: Common European Framework of Reference for Languages
CI: Confidence interval
OR: Odds ratio
PHQ-8: Patient Health Questionnaire Depression Scale
